# Time evolution of Fukushima-derived radiocesium in the western subtropical gyre of the North Pacific Ocean by 2017

**DOI:** 10.1007/s10967-018-6133-5

**Published:** 2018-08-21

**Authors:** Yuichiro Kumamoto, Michio Aoyama, Yasunori Hamajima, Eitarou Oka, Akihiko Murata

**Affiliations:** 1 Research and Development Center for Global Change, Japan Agency for Marine-Earth Science and Technology, 2-15 Natushima-cho, Yokosuka, Kanagawa 237-0061 Japan; 2 Institute of Environmental Radioactivity, Fukushima University, 1-1 Kanayagawa, Fukushima, Fukushima 960-1296 Japan; 3 Low Level Radioactivity Laboratory, Kanazawa University, Wake, Nomi, Ishikawa 923-1224 Japan; 4 Atmosphere and Ocean Research Institute, The University of Tokyo, 5-1-5 Kashiwanoha, Kashiwa, Chiba 277-8564 Japan

**Keywords:** Fukushima Dai-ichi Nuclear Power Plant accident, Radiocesium, North Pacific Ocean, Western subtropical gyre

## Abstract

In 2015–2017, we measured activity concentration of radiocesium in the western subtropical gyre of the North Pacific Ocean and revealed the time evolution of radiocesium derived from the Fukushima Dai-ichi Nuclear Power Plant (FNPP1) accident between 2011 and 2017. The FNPP1-derived radiocesium deposited on the area south of the Kuroshio/Kuroshio Extension Currents in March 2011 was transported southward and westward through subsurface layers due to subduction of the subtropical mode water. In 2014, the radiocesium in the subsurface layers returned to the north and circulated within the Kuroshio recirculation area. Then in 2015–2017, the radiocesium re-circulated with the area.

**Electronic supplementary material:**

The online version of this article (10.1007/s10967-018-6133-5) contains supplementary material, which is available to authorized users.

## Introduction

The massive Tohoku earthquake and consequent giant tsunamis on 11 March 2011 resulted in serious damage to the Fukushima Dai-ichi Nuclear Power Plant (FNPP1) in eastern Japan. Radiocesium (^134^Cs and ^137^Cs) released from the damaged FNPP1 caused radioactive contamination in the land of eastern Japan and the North Pacific Ocean mostly in March and April 2011 [1]. Measurements of ^134^Cs and ^137^Cs activity concentrations in soil collected in Japan revealed that (1) the activities of ^134^Cs and ^137^Cs released from the FNPP1 were equivalent at a 1:1 ratio approximately [2] and (2) the total deposition of ^134^Cs (or ^137^Cs) activity on the land was 2.4 PBq (10^15^ Bq) [3]. Recent estimates of the total ^134^Cs (or ^137^Cs) deposition in the ocean tend to converge on a range of 10–20 PBq [4]. ^134^Cs (or ^137^Cs) was also discharged directly into the North Pacific due to leakage of contaminated water from the FNPP1, which was estimated to be a range of 2–6 PBq [4].

Before the FNPP1 accident, radiocesium was also released into the North Pacific by atmospheric nuclear weapons testing mainly in the 1950s and 1960s [5]. The bomb-derived ^137^Cs deposited on the North Pacific remained in the ocean in March 2011 because of its long half-life (30.17 years). After March 2011, the FNPP1-derived ^137^Cs was added to the bomb-derived ^137^Cs, which resulted in about 30% increase of ^137^Cs activity in the North Pacific [6]. In contrast, the ^134^Cs released before the FNPP1 accident had disappeared, because its half-life is only 2.06 years. Therefore, ^134^Cs is an unequivocal indicator of the radiocesium contamination due to the FNPP1 accident.

**Fig. 1:**
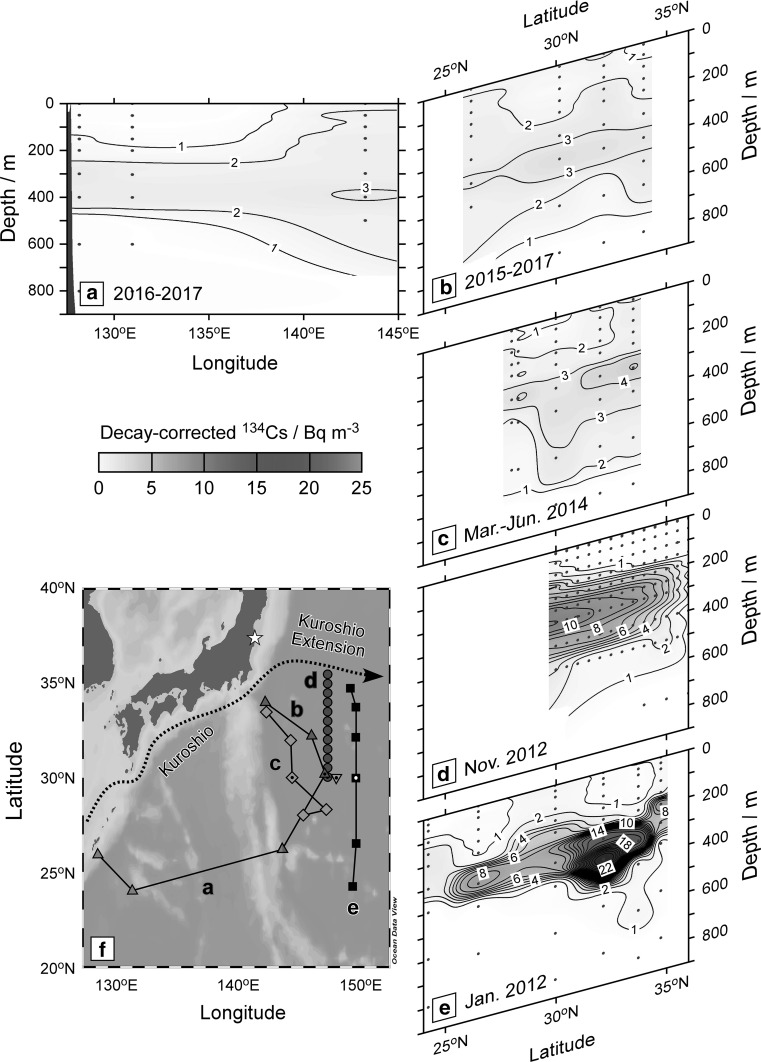
Transections of ^134^Cs activity concentration (Bq m^−3^) along approximately 25°N in 2016–2017 (**a**) and approximately 145°E in 2015–2017 (**b**). Those along approximately 145°E in March–June 2014 (**c**) [16], 147°E in November 2012 (**d**) [13], and 149°E in January 2012 (**e**) [11] are also shown. The activity concentration of ^134^Cs was corrected to the FNPP1 accident date. The contour interval is 1 Bq m^−3^. Dots are sampling depths at each station. The locations of the sampling stations in a/b (triangles), c (diamonds), d (circles), and e (squares) are shown in the map (f). The inverted triangle indicates a sampling station in April 2013 [9]. The vertical profiles at stations marked with a dot (30°N) are shown in Fig. 2. The star and dotted arrow indicate the FNPP1 location and schematic stream lines of the Kuroshio and Kuroshio Extension Current, respectively. This figure was drawn using Ocean Data View software [23]

The FNPP1 (37.4°N/141°E) is situated to the north of the Kuroshio Extension Current around 35°N, which corresponds to the Kuroshio Front (Fig. 1f). The FNPP1-derived ^134^Cs directly-discharged and atmospheric-deposited north of the Kuroshio Front was transported eastward along surface currents. In summer 2012, about one and half years after the accident, a water mass with high activity concentration of ^134^Cs was observed around 165°E–170°W between 40°N and 50°N in surface layers [7]. This high-^134^Cs water was then reached to stations in the Gulf of Alaska in 2015 [8].

The area south of the Kuroshio Front, namely the western subtropical gyre, the activity concentration of ^134^Cs in surface water was lower than that in the area north of the front, because the Kuroshio Front restricted surface water exchange across it [7]. In the western subtropical gyre, however, subsurface maxima of ^134^Cs (> 10 Bq m^−3^) in approximately 200–600 m depth had been observed since several months after the accident [9–13]. The subsurface layer of the ^134^Cs maximum agrees with density layers of the subtropical mode water (STMW) [14] in the North Pacific Ocean. Potential water-density anomaly (σ_θ_) defined by [potential water density (kg m^−3^) − 1000] of STMW ranges about 25.0–25.6 kg m^−3^. STMW is formed just south of the Kuroshio and Kuroshio Extension Currents in the mid-winter due to severe cooling by cold monsoon wind and then transported southward and westward through subsurface layers [15]. Therefore, it was concluded that ^134^Cs deposited just south of the Kuroshio and Kuroshio Extension Currents in March 2011 was conveyed southward through the subsurface layers due to the formation and subduction of STMW.

We measured vertical profiles of radiocesium in the western subtropical gyre of the North Pacific and concluded that the FNPP1-derived radiocesium had spread throughout the whole area of the western subtropical area by 2014 [16]. Following the observations in 2014, we continued to measure radiocesium concentration in the western subtropical gyre in 2015, 2016, and 2017. We compiled radiocesium data from this study and previous works and revealed time evolution of the FNPP1-derived radiocesium in the western subtropical gyre by 2017.

## Experimental

### Samples

Seawater samples (10 or 20 L) for radiocesium measurements were collected at 6 stations between October 2015 and January 2017 during five research cruises of “Hakuho-maru” (KH-16-3), “Kaimei” (KM16-08 and KM17-01), “Keifu-maru” (KS16-09), and “Shinsei-maru” (KS-15-14) (Fig. 1f). Surface seawater was collected using a bucket, a 12-l Niskin sampling bottle, or a pump for surface water. Seawater samples from deep layers were collected using the Niskin bottles equipped to a carousel multi-sampling system with sensors (Model SBE 9 plus/11 plus, Seabird Electronics Inc.) which measured salinity, temperature, and pressure. All the samples were acidified by adding concentrated nitric acid. Because we did not filter the seawater sample, measured radiocesium in the seawater sample included those in dissolved and particulate fractions. The particulate fraction, however, is expected to be negligible because of soluble nature of cesium [17].

### Measurements

After the cruises, radiocesium in the seawater sample was concentrated onto ammonium phosphomolybdate (AMP) for measurement of gamma-ray activity [18] in our onshore laboratory of the Mutsu Institute for Oceanography, Japan Agency for Marine-Earth Science and Technology (MIO/JAMSTEC). The radiocesium activity concentration in the AMP/Cs compound was measured using gamma-ray spectrometers in MIO/JAMSTEC. To remove ^40^K that is a major interfering substance, radiocesium in the AMP/Cs compound of the KS16-09 sample was refined into a platinate salt using a precipitation method [18] and re-measured using low-background gamma-ray spectrometers in Low Level Radioactivity Laboratory, Kanazawa University (LLRL/KU) [19]. The detection limit of ^134^Cs (^137^Cs) decay-corrected to the accident date (11 March 2011) for the measurements in LLRL/KU and MIO/JAMSTEC were calculated to be approximately 0.2 (0.05) and 0.4 (0.1) Bq m^−3^, respectively. All the radiocesium and hydrographic data are listed in the supplementary table.

## Results

### Vertical distribution in 2015–2017

In 2015–2017, the subsurface maximum of ^134^Cs still remained in 200–600 m depth in the western subtropical gyre of the North Pacific, south of the Kuroshio and Kuroshio Extension Currents (Fig. 1a, b). The concentrations in 200–600 m depth (1.0–2.9 Bq m^−3^) at 128°E and 131°E in November 2016 were lower than those (2.1–3.2 Bq m^−3^) at 143°E in January 2017 (Fig. 1a). Because the sampling at the stations conducted simultaneously, the observed difference in vertical profile of ^134^Cs between the western and eastern stations suggests spatial variation. ^134^Cs profile at stations along approximately 145°E were similar: the maximum concentrations (3.2–3.8 Bq m^−3^) appeared at 400 m depth (Fig. 1b), which suggests its small temporal variation during the sampling period from October 2015 to January 2017.

### Temporal change in vertical distribution

To discuss the temporal change by 2017, vertical distributions of ^134^Cs from 2012 to 2014 in the area south of the Kuroshio Front between 140°E and 150°E are also shown in Fig. 1. In March–June 2014 (Fig. 1c), the subsurface maxima of ^134^Cs were shallower (about 300 m depth) than those in 2015–2017 (about 400 m depth). The peak concentrations (4.5–5.1 Bq m^−3^) at the two northern stations (33.5°N and 32.0°N) were higher than those (3.1–4.4 Bq m^−3^) observed in the three southern stations (30.0°N, 28.3°N, and 28.0°N). In November 2012 (Fig. 1d), the subsurface maxima of ^134^Cs around 300 m depth were more apparent and the peak concentrations were more than three times higher than those in 2014. The peak concentration was higher in the south and highest at the southernmost station (13.1 Bq m^−3^). In January 2012 (Fig. 1e), the peak concentration of the subsurface maximum was highest at 370 m depth at 32.2°N (27.4 Bq m^−3^) and lower in the south.

**Fig. 2:**
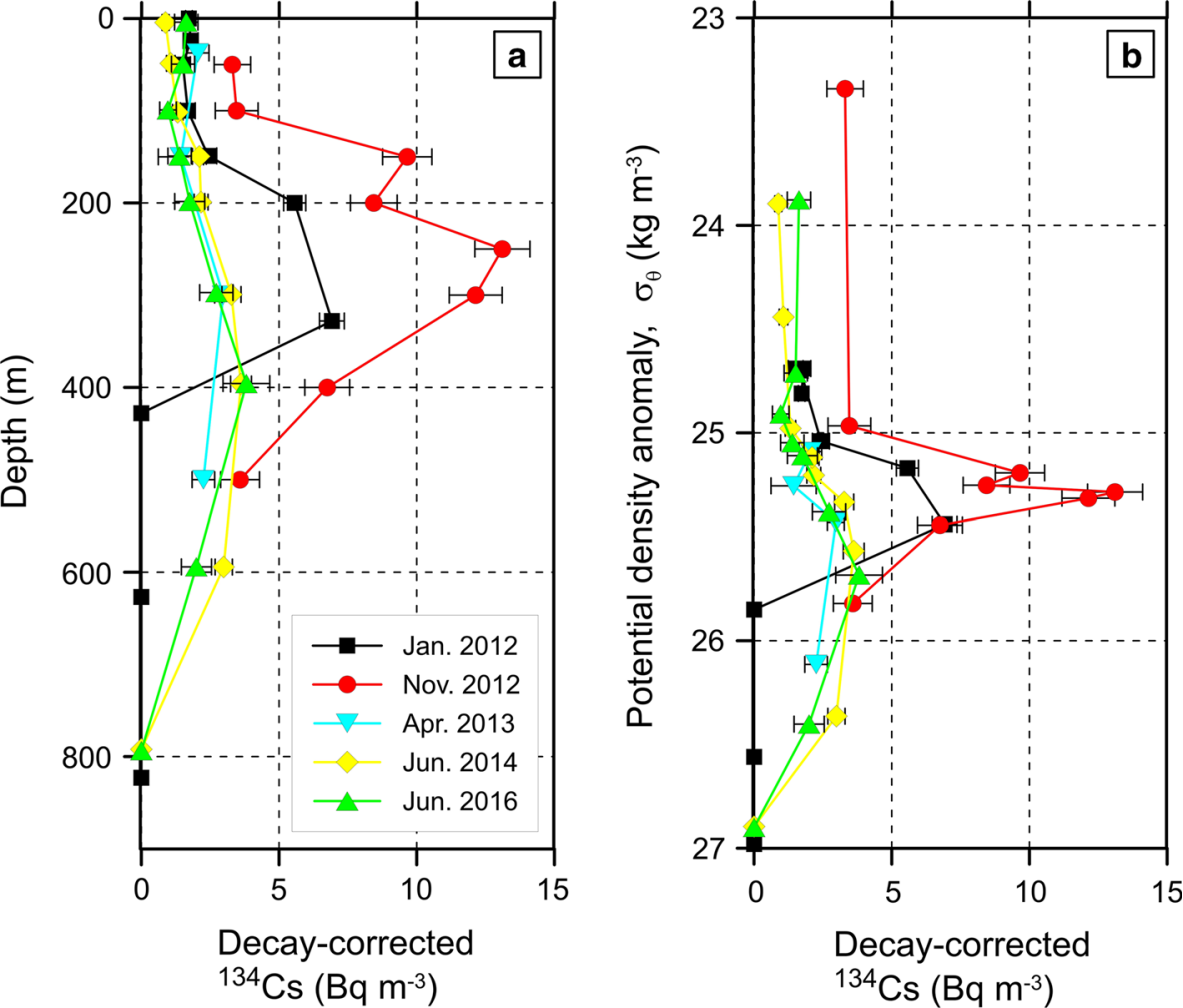
**a** Vertical profiles of activity concentrations of ^134^Cs (Bq m^−3^) against water depth (m) at stations around 30°N in January 2012 (squares, [11]), November 2012 (circles, [13]), April 2013 (inverted triangles, [9]), June 2014 (diamonds, [16]), and June 2016 (triangles). The activity concentration of ^134^Cs was corrected to the FNPP1 accident date. The sampling locations are shown in Fig. 1f. **b** Same as (**a**) but for those against potential density anomaly (σ_θ_, kg m^−3^)

Vertical distribution of ^134^Cs at 30°N between January 2012 and June 2016 are shown in Fig. 2a. The peak concentration of the subsurface maximum increased about twice between January and November 2012. Then in April 2013, the peak concentration decreased in about half of that observed in January 2012. In June 2014, the vertical profile was similar with that observed in April 2013. The temporal change during 2 years between June 2014 and June 2016 was also small. The subsurface maximum observed in 2012 appeared in isopycnal layers of about 25.0–25.6 σ_θ_ (kg m^−3^), namely in STMW (Fig. 2b). After 2013, the subsurface maximum deepened to layers of about 25.4–26.0 σ_θ_ (kg m^−3^).

### Temporal change in vertical inventory

**Fig. 3:**
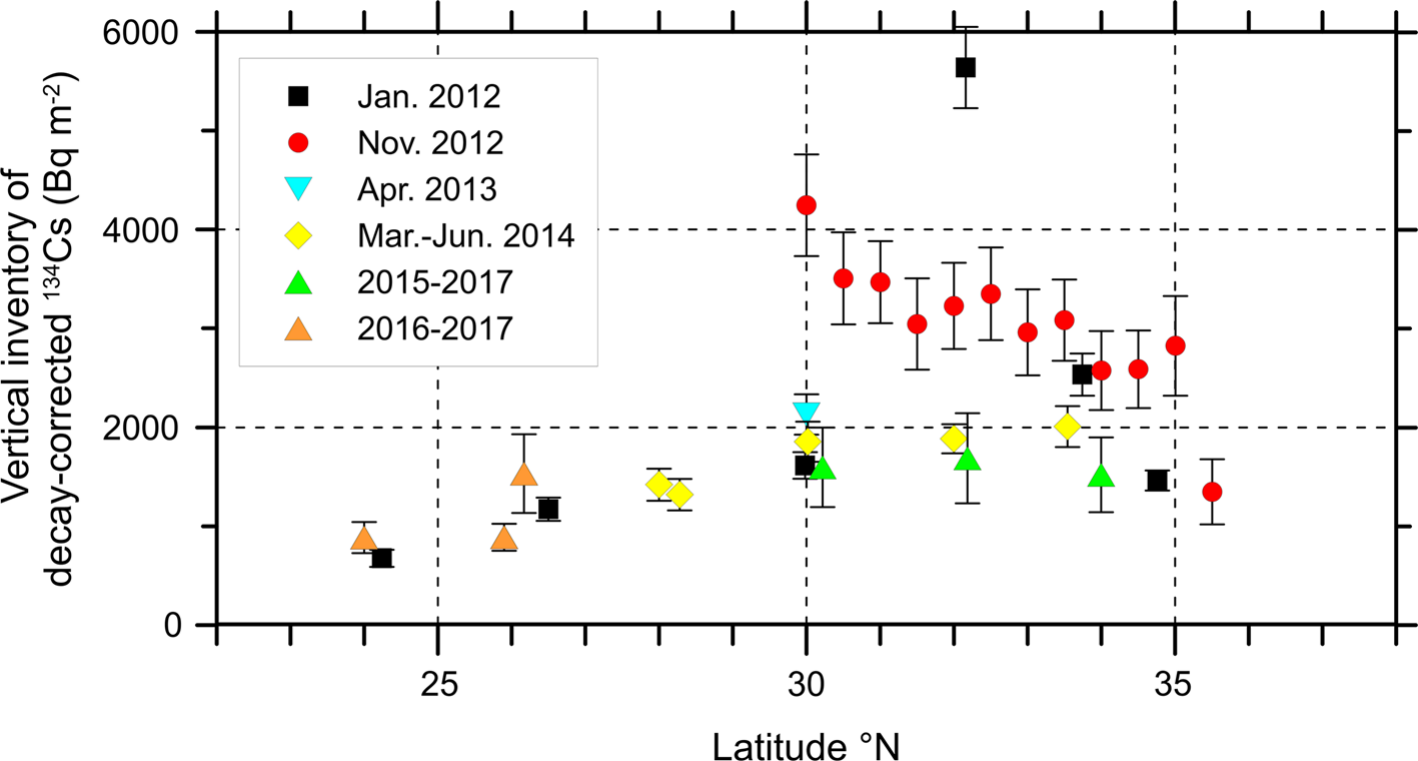
Distribution of vertical inventories from surface to 800 m depth of decay-corrected ^134^Cs (Bq m^−2^) along latitude in January 2012 (squares, [11]), November 2012 (circles, [13]), April 2013 (inverted triangles, [9]), March–June 2014 (diamonds, [16]), and 2015–2017 (triangles). The sampling locations are shown in Fig. 1f

Vertical inventory of ^134^Cs (Bq m^−2^) decay-corrected between surface and 800 m depth was determined by simple trapezoidal integration of activity concentrations (Bq m^−3^) versus depth (m). At 30°N, the vertical inventory increased from 1620 ± 130 Bq m^−2^ in January 2012 to 4250 ± 520 Bq m^−2^ in November 2012 (Fig. 3). Then it reduced to 2130 ± 200 Bq m^−2^ in April 2013. Similar inventories of 1860 ± 200 Bq m^−2^ and 1600 ± 400 Bq m^−2^ were observed in June 2014 and June 2016, respectively. In January 2012, the largest inventory was observed at 32°N (5640 ± 410 Bq m^−2^). In November 2012, the inventory between 30°N and 35°N increased to 2570–4250 Bq m^−2^ except those around 32°N and became larger toward south. In 2014, the inventory decreased less than 2000 Bq m^−2^. The inventories at the northern three stations (1860–2000 Bq m^−2^), however, significantly larger than those at the southern two stations (1320–1420 Bq m^−2^). In 2015–2017, the inventories at the southern or western (approximately 130°E) two stations were significantly smaller than those at the eastern stations (approximately 145°E).

## Discussion

In January 2012, about 10 months after the FNPP1 accident, a main body of the subsurface maximum remained between 30°N and 35°N (Fig. 1e), where STMW was formed in March 2011, just after the accident. In November 2012, about 20 months after the accident, the main body was probably transported to the south of 30°N (Fig. 1d). In March–June 2014, about 40 months after the accident, the highest peak concentration of the subsurface maximum appeared again between 30°N and 35°N (Fig. 1c), which implies a return of the main body of the high concentration water to the north along the anti-cyclonic circulation of STMW in the western subtropical gyre [20]. The small spatial and temporal variations in the vertical profiles of ^134^Cs in 2015–2017 (Fig. 1b) suggest that the peak concentration in the subsurface layer was mixed horizontally possibly due to re-circulation of STMW. The deepening of the peak concentration (Fig. 2) could be explained by diapycnal vertical mixing and erosion of the upward spreading due to newly-formed STMW after the winter of 2012, whose ^134^Cs concentration was lower than that in STMW subducted in March 2011 [16].

**Fig. 4:**
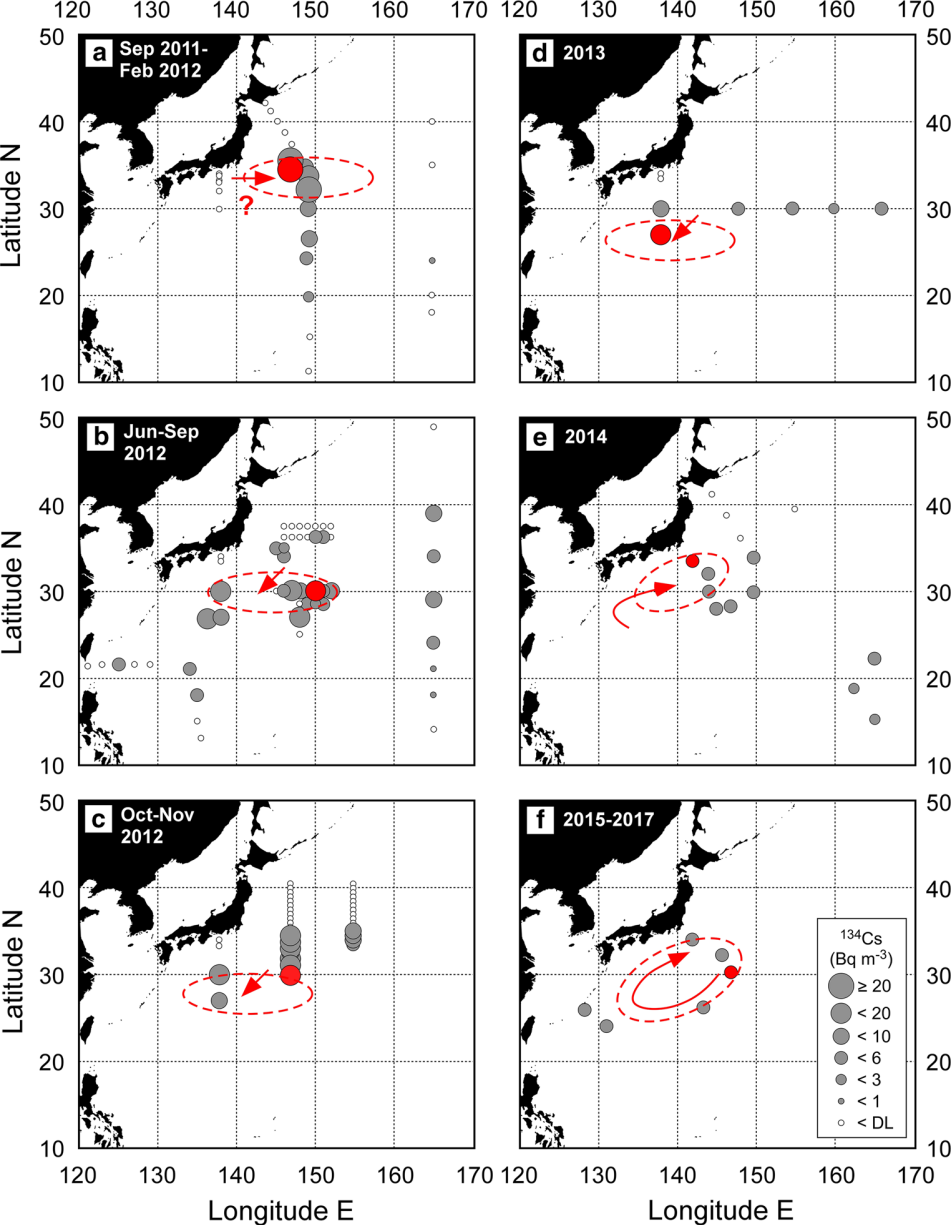
Activity concentration of ^134^Cs (Bq m^−3^) decay-corrected to the FNPP1 accident date in subsurface layer between 25.0 and 25.6 potential density anomaly (σ_θ_, kg m^−3^) in September 2011–February 2012 (**a**), June–September 2012 (**b**), October–November 2012 (**c**), 2013 (**d**), 2014 (**e**), and 2015–2017 (**f**). Open small circles show concentration below the detection limit. The red circle and area within broken line in each period indicate the observed highest concentration and speculated area of the main body of the high concentration in the subsurface layer, respectively. This figure was drawn using Ocean Data View software [23]

To confirm the circulation and re-circulation of the FNPP1-derived ^134^Cs in the STMW layer, we complied ^134^Cs data in the subsurface layer (25.0–25.6 kg m^−3^) in the western subtropical gyre between 2011 and 2017 (Fig. 4) [9–13, 21]. Between September 2011 and February 2012 (Fig. 4a), the subsurface maximum was apparent only at stations along 150°E and north of 20°N. The main body of the high concentration water, which was formed between 30°N and 35°N approximately in March 2011, remained in the same latitude range about 10 months later. This apparent stagnation of the main body implies that eastward transport was predominant in this early stage. In June–September 2012 (Fig. 4b), the subsurface maximum spread to the west (125°E), east (165°E), and south (18°N), namely into almost the whole western subtropical gyre. The main body was transported to around 30°N due to southward subduction of STMW. In October–November 2012 (Fig. 4c), it was continuously transported southwestward and reached to the south of 30°N in 2013 (Fig. 4d). In 2014 (Fig. 4e), the main body returned to the north at the western edge of the western subtropical area and then moved eastward along the northern rim of STMW circulation. During 2015–2017 (Fig. 4f), it re-circulated within an area south of Japanese islands that corresponds to the Kuroshio recirculation area. As a result, the small spatial and temporal variations in the vertical profile and inventory were observed within the area (Figs. 1b, 3). Approximate 3 years of the circulation time of STMW in the Kuroshio recirculation area, which was derived from the time evolution of the subsurface ^134^C maximum, agrees with the renewal time of STMW in the western subtropical area, 2–9 years [22].

## Conclusions

We revealed the time evolution of FNPP1-derived radiocesium in the western subtropical gyre between 2011 and 2017. In March 2011, the radiocesium was released from the damaged FNPP1 and some of that was deposited on the area south of the Kuroshio/Kuroshio Extension Currents, where STMW was sinking to the subsurface layer. Along the southward and westward transport of STMW, the FNPP1-derived radiocesium was transported to the south of 30°N by 2013 through the subsurface layer. Then the subsurface maximum returned to the north and circulated in the Kuroshio recirculation area by 2014. It re-circulated within the area in 2015–2017. The circulation time of STMW in the Kuroshio recirculation area was estimated to be about 3 years.

## Electronic supplementary material

Below is the link to the electronic supplementary material.

Supplementary material 1 (XLSX 22 kb)

## References

[1] Yoshida N, Kanda J (2012). Tracking the Fukushima radionuclides. Science.

[2] Saito K, Tanihata I, Fujiwara M, Saito T, Shimoura S, Otsuka T, Onda Y, Hoshi M, Ikeuchi Y, Takahashi F, Kinouchi N, Saegusa J, Seki A, Takemiya H, Shibata T (2015). Detailed deposition density maps constructed by large-scale soil sampling for gamma-ray emitting radioactive nuclides from the Fukushima Dai-ichi Nuclear Power Plant accident. J Environ Radioact.

[3] Morino Y, Ohara T, Watanabe M, Hayashi S, Nishizawa M (2013). Episode analysis of deposition of radiocesium from the Fukushima Daiichi Nuclear Power Plant accident. Environ Sci Technol.

[4] Buesseler K, Dai M, Aoyama M, Benitez-Nelson C, Charmasson S, Higley K, Maderich V, Masqué P, Morris PJ, Oughton D, Smith JN (2017). Fukushima Daiichi–derived radionuclides in the ocean: transport, fate, and impacts. Annu Rev Mar Sci.

[5] Aoyama M, Hirose K, Igarashi Y (2006). Re-construction and updating our understanding on the global weapons tests ^137^Cs fallout. J Environ Monit.

[6] Aoyama M, Kajino M, Tanaka TY, Sekiyama TT, Tsumune D, Tsubono T, Hamajima Y, Inomata Y, Gamo T (2016). ^134^Cs and ^137^Cs in the North Pacific Ocean derived from the March 2011 TEPCO Fukushima Dai-ichi Nuclear Power Plant accident, Japan. Part two: estimation of ^134^Cs and ^137^Cs inventories in the North Pacific Ocean. J Oceanogr.

[7] Kumamoto Y, Aoyama M, Hamajima Y, Nishino S, Akihiko Murata A, Kikuchi T (2016). Meridional distribution of Fukushima-derived radiocesium in surface seawater along a trans-Pacific line from the Arctic to Antarctic Oceans in summer 2012. J Radioanal Nucl Chem.

[8] Smith JN, Rossi V, Buesseler KO, Cullen JT, Cornett J, Nelson R, Macdonald AM, Robert M, Kellogg J (2017). Recent transport history of Fukushima radioactivity in the Northeast Pacific Ocean. Environ Sci Technol.

[9] Yoshida S, Macdonald AM, Jayne SR, Rypina II, Buesseler KO (2015). Observed eastward progression of the Fukushima ^134^Cs signal across the North Pacific. Geophys Res Lett.

[10] Aoyama M, Hamajima Y, Hult M, Uematsu M, Oka E, Tsumune D, Kumamoto Y (2016). ^134^Cs and ^137^Cs in the North Pacific Ocean derived from the March 2011 TEPCO Fukushima Dai-ichi Nuclear Power Plant accident, Japan. Part one: surface pathway and vertical distributions. J Oceanogr.

[11] Kumamoto Y, Aoyama M, Hamajima Y, Aono T, Kouketsu S, Murata A, Kawano T (2014). Southward spreading of the Fukushima-derived radiocesium across the Kuroshio Extension in the North Pacific. Sci Rep.

[12] Kaeriyama H, Shimizu Y, Ambe D, Masujima M, Shigenobu Y, Fujimoto K, Ono T, Nishiuchi K, Taneda T, Kurogi H, Setou T, Sugisaki H, Ichikawa T, Hidaka K, Hiroe Y, Kusaka A, Kodama T, Kuriyama M, Morita H, Nakata K, Morinaga K, Morita T, Watanabe T (2014). Southwest intrusion of ^134^Cs and ^137^Cs derived from the Fukushima Dai-ichi Nuclear Power Plant accident in the western North Pacific. Environ Sci Technol.

[13] Kaeriyama H, Shimizu Y, Setou T, Kumamoto Y, Okazaki M, Ambe D, Ono T (2016). Intrusion of Fukushima-derived radiocaesium into subsurface water due to formation of mode waters in the North Pacific. Sci Rep.

[14] Masuzawa J (1969). Subtropical mode water. Deep Sea Res.

[15] Suga T, Hanawa K (1995). The subtropical mode water circulation in the North Pacific. J Phys Oceanogr.

[16] Kumamoto Y, Aoyama M, Hamajima Y, Nagai H, Yamagata T, Kawai Y, Oka E, Yamaguchi A, Imai K, Murata A (2017). Fukushima-derived radiocesium in the western North Pacific in 2014. J Radioanal Nucl Chem.

[17] Ramzaev V, Nikitin A, Sevastyanov A, Artemiev G, Bruk G, Ivanov S (2014). Shipboard determination of radiocesium in seawater after the Fukushima accident: results from the 2011–2012 Russian expeditions to the Sea of Japan and western North Pacific Ocean. J Environ Radioact.

[18] Aoyama M, Hirose K, Povinec PP (2008). Radiometric determination of anthropogenic radionuclides in seawater. Analysis of environmental radionuclides, radioactivity in the environment.

[19] Hamajima Y, Komura K (2004). Background components of Ge detectors in Ogoya underground laboratory. Appl Radiat Isot.

[20] Oka E, Qiu B (2012). Progress of North Pacific mode water research in the past decade. J Oceanogr.

[21] Men W, He J, Wang F, Wen Y, Li Y, Huang J, Yu X (2015). Radioactive status of seawater in the northwest Pacific more than one year after the Fukushima nuclear accident. Sci Rep.

[22] Suga T, Aoki Y, Saito H, Hanawa K (2008). Ventilation of the North Pacific subtropical pycnocline and mode water formation. Prog Oceanogr.

[23] Schlitzer R, Ocean data view. http://odv.awi.de. Accessed 2 Aug 2018

